# MetaboSense: An Integrated Quantum Dot‐Mediated Aptamer Assay and Modular Microfluidic Platform for Continuous In‐Line Monitoring of Lung Metabolism During *Ex Vivo* Perfusion

**DOI:** 10.1002/advs.77123

**Published:** 2026-08-13

**Authors:** Sanjana Srikant, Pouyan Keshavarz Motamed, Thomas Borrillo, Lielle Ronen, Gabriel Siebiger, Guillermo Garza, Hesam Abouali, Md Fahim Al Fattah, Ines Jaber, Dayan Ban, Marcelo Cypel, Mingyao Liu, Shaf Keshavjee, Andrew T. Sage, Mahla Poudineh

**Affiliations:** ^1^ Department of Electrical and Computer Engineering University of Waterloo Waterloo Ontario Canada; ^2^ Waterloo Institute For Nanotechnology University of Waterloo Waterloo Ontario Canada; ^3^ Latner Thoracic Research Laboratories Toronto General Hospital University Health Network Toronto Ontario Canada; ^4^ Toronto Lung Transplant Program Ajmera Transplant Centre University Health Network Toronto Ontario Canada; ^5^ Institute of Medical Science Temerty Faculty of Medicine University of Toronto Toronto Ontario Canada; ^6^ Department of Surgery Temerty Faculty of Medicine University of Toronto Toronto Ontario Canada

**Keywords:** continuous monitoring, ex vivo lung perfusion, glucose, in‐line measurement, lactate, metabolites

## Abstract

Organ transplantation provides a life‐saving intervention for patients with end‐stage lung disease; however, access to this treatment remains limited by the shortage of donor organs. *Ex vivo* lung perfusion (EVLP) is a platform that enables surgeons to assess extended‐criteria for donor lungs prior to transplantation and has safely expanded the donor pool. Yet current EVLP biochemical assessments rely on intermittent manual sampling and fail to capture dynamic metabolic changes. Here we present MetaboSense, a first‐of‐its‐kind microfluidic platform for continuous, real‐time, multiplexed monitoring of glucose and lactate—key metabolites that report on donor lung metabolism—during EVLP. The system integrates a bead‐based, quantum dot‐mediated aptamer assay with modular microfluidics for in‐line biomarker measurement. MetaboSense achieves limits of detection of 0.74 mM (glucose) and 1.19 mM (lactate) with analytical precision (R^2^ > 0.97). Clinical validation across eleven EVLP cases demonstrates strong correlation with gold‐standard arterial blood gas measurements. In‐line deployment within an EVLP circuit during perfusion of porcine lungs confirms MetaboSense's ability to track metabolite fluctuations with 60‐s temporal resolution. This platform eliminates labor‐intensive sampling and provides unprecedented granularity in lung metabolomics during EVLP, representing an important step toward real‐time EVLP analytics and improved organ selection and transplant outcomes.

## Graphical abstract

MetaboSense is a first‐of‐its‐kind microfluidic platform enabling continuous, in‐line monitoring of glucose and lactate during *ex vivo* lung perfusion. Combining a quantum dot–mediated aptamer assay with mixing, depletion, and optofluidic detection modules, it achieves 60–s temporal resolution and shows strong agreement with standard blood gas analyzers, providing high‐resolution metabolic data that could support donor lung evaluation in the future.

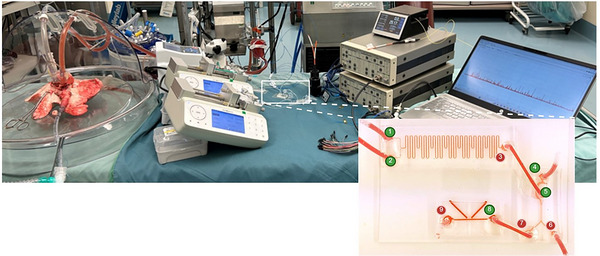

## 1 Introduction

**FIGURE 1:**
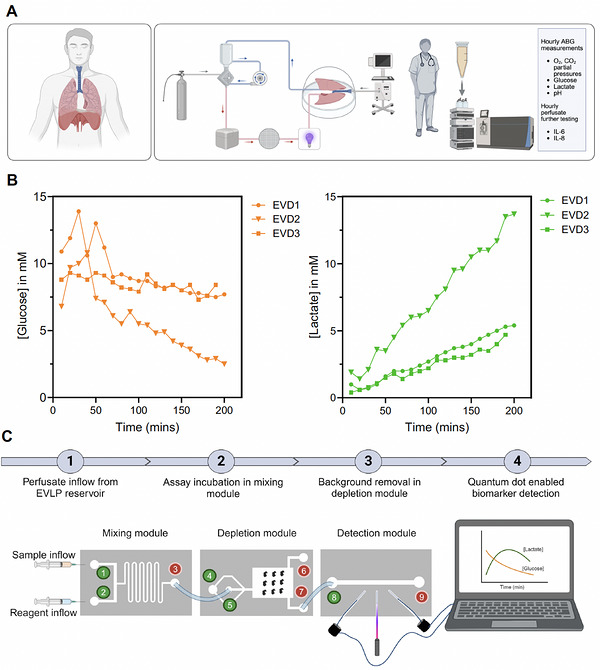
Overview of conventional lung monitoring during EVLP and the MetaboSense platform. (A) Schematic depicting EVLP protocol. (B) Graphs showing the trends of glucose and lactate measured across 3 h of EVLP with perfusate samples taken every 10 min in three donor lungs and measured on‐site; EVD1 and 2 show measurements from lungs that were rejected for transplant; EVD3 shows the measured values from a lung accepted for transplant. Measurements were performed using gold‐standard arterial blood gas measurements. (C) MetaboSense platform designed for continuous and real‐time measurements of glucose and lactate during EVLP. Microfluidic schematics of each module are shown. Inlets and outlets for each module are numbered, with inlets labeled in green and outlets in red. Inlets 1 and 2 deliver perfusate samples and reagents to the mixing module; inlet 4 supplies sheath buffer to the depletion module; inlet 5 receives the mixed perfusate and reagents from the mixing module; and inlet 8 transfers beads from the depletion module to the detection module. Outlet 3 carries the mixed solution from the mixing to the depletion module; outlet 6 removes waste (unbound reagents) from the depletion module; outlet 7 directs separated assay beads to the detection module; and outlet 9 is the final output of the detection module.

Lung transplantation is a life‐saving treatment for patients with end‐stage pulmonary disease. However, its clinical application is constrained by the limited availability of suitable donor organs. Approximately 82% of lungs considered for transplantation are ultimately discarded, with poor organ function at initial assessment being a key reason for declining donor lungs [1, 2, 3, 4, 5, 6, 7, 8, 9, 10, 11, 12, 13]. The advent of normothermic *ex vivo* lung perfusion (EVLP) has expanded donor lung utilization by allowing surgeons an opportunity to comprehensively assess and potentially rehabilitate extended‐criteria donor lungs that may have otherwise been considered unsuitable for transplantation [3, 4, 6, 8, 14, 15, 16]. EVLP facilitates lung assessment in a controlled, physiological environment, significantly increasing the donor pool without compromising short‐term or long‐term transplantation outcomes [4, 15, 17, 18]. Particularly, by utilizing an EVLP circuit equipped with an intensive care unit (ICU)‐grade ventilator to recapitulate normal lung function (Figure 1A), donor lungs initially deemed unsuitable for transplantation have been successfully recovered more frequently [19, 20].

During standard clinical EVLP, human donor lungs are ventilated and perfused with an acellular solution at 37°C for up to 6 h. During this period, physiological, biochemical, and inflammatory parameters—such as airway pressures, lung compliances, as well as perfusate blood gases, pH, cytokine levels, and cellular metabolites—are measured [4, 5, 7, 8, 9, 11, 14]. The biochemical measurements traditionally rely on periodic (i.e., hourly) manual sampling of perfusate and subsequent analysis with a blood gas analyzer [12]. These discrete, labor‐intensive assessments provide only snapshots of lung health, potentially missing critical transient changes in lung function that occur between sampling intervals. Improving the temporal resolution of biomarker monitoring during EVLP presents an opportunity to capture the dynamics of biological changes and more accurately characterize lung trajectories, which may enhance organ rehabilitation and selection. In critical care settings, leveraging high‐frequency continuous data modalities has similarly been shown to improve predictive modeling of clinical outcomes and enable earlier detection of clinical deterioration [21, 22].

Metabolic profiling during EVLP offers critical insight into lung viability, with glucose and lactate among the most informative and routinely monitored biomarkers. During the *ex vivo* perfusion, lungs consume glucose and produce lactate. In normal human lungs assessed on EVLP, glucose levels typically decline from 9 to 6 mM, while lactate levels increase up to around 10 mM [17]. Increased glucose consumption has been associated with impaired lung function, including worse oxygenation and increased pulmonary edema, highlighting its importance as a biomarker for evaluating lung quality during EVLP [23]. Recent predictive modeling studies conducted in EVLP‐assessed lungs have underscored the clinical utility of glucose and lactate dynamics, identifying these biomarkers as important predictors of transplant viability and post‐transplant outcomes [5, 6, 7]. Importantly, some studies in real‐world settings indicate that the predictive value of glucose consumption and lactate production changes over the course of EVLP, implying that crucial information may be missed between hourly measurements [24].

Various technologies have been developed for continuous glucose and lactate monitoring. For example, commercially available continuous glucose monitors used in clinical diabetes management employ macroelectrodes functionalized with glucose oxidase to track glucose levels within interstitial fluid [25]. While dedicated commercial technologies for continuous lactate monitoring remain limited, several platforms have been reported that enable lactate measurement in interstitial fluid using either lactate oxidase or lactate‐specific aptamer probes [26, 27]. Despite these advancements, continuous glucose and lactate monitoring has yet to be translated into ex vivo organ perfusion systems.

Recent reports highlight the need for continuous metabolite monitoring during EVLP [28]. One study leveraged microdialysis probes to characterize the metabolic profile of interstitial fluid through benchtop analysis of glucose, lactate, pyruvate, and glutamate levels. This work demonstrates that metabolomic profiling enables the discrimination of donor lungs likely to have an unfavorable clinical outcome [28]. Although this study demonstrates the importance of frequent metabolite monitoring during lung perfusion, the ability to continuously monitor metabolites in the EVLP perfusate using an in‐line sensor has yet to be achieved.

We have recently developed a specially designed microfluidic system for continuous, real‐time biomarker monitoring. This system integrates three distinct microfluidic modules, each designed to replicate a specific step of a traditional benchtop biochemical assay (Figure 1C). Previously, we employed this integrated microfluidic platform for real‐time detection of insulin and glucagon in live rats [29]. In the present study, we tailored this microfluidic system for real‐time and continuous measurement of glucose and lactate, leveraging bio‐affinity aptamer probes conjugated with quantum dots as fluorescence tags. This novel EVLP sensor, termed “MetaboSense,” was validated through examinations of the individual microfluidic modules and the fully integrated assay platform, followed by continuous and multiplexed measurements of spiked glucose and lactate in perfusate samples. The system was then tested using perfusate samples collected from eleven clinical cases, and the glucose and lactate levels measured by MetaboSense were compared with the ones obtained from gold‐standard arterial blood gas measurements. Finally, we deployed and examined the in‐line capability of MetaboSense for in‐line measurements by connecting the system during EVLP. These results demonstrate the potential of MetaboSense for continuous and in‐line measurement of glucose and lactate, eliminating the need for manual sample collection and off‐site analysis.

## 2 Results

### 2.1 Current Clinical Challenges Associated With Infrequent Sampling During EVLP

To underscore the need for continuous monitoring of glucose and lactate, we investigated glucose and lactate trends in lungs during EVLP using a high‐frequency manual sampling protocol. Compared to conventional hourly measurements, perfusate was sampled every 10 min in three clinical EVLP cases, and glucose and lactate levels were measured using traditional arterial blood gas measurements (Figure 1B). We observed unique trends in glucose and lactate for each lung, with glucose rapidly fluctuating in the two lungs (Figure 1B‐EVD1 and EVD2) that were rejected for transplantation, while it more steadily decreased in the healthy lung accepted for transplant (Figure 1B‐EVD3). These results, and previously reported studies where glucose and lactate are measured infrequently, demonstrate that there is a largely underexplored aspect of glucose and lactate dynamics during EVLP [17, 24]. Conventional hourly measurements leave clinically relevant data on lung function untapped, limiting the evaluation and potential clinical intervention on lungs that are being assessed by EVLP [3, 4, 6, 17, 24]. Taken together, the development of an inline sensor capable of high‐frequency measurements addresses a key unmet need in EVLP systems.

### 2.2 Modular Characterization of the MetaboSense Microfluidic Platform

**FIGURE 2:**
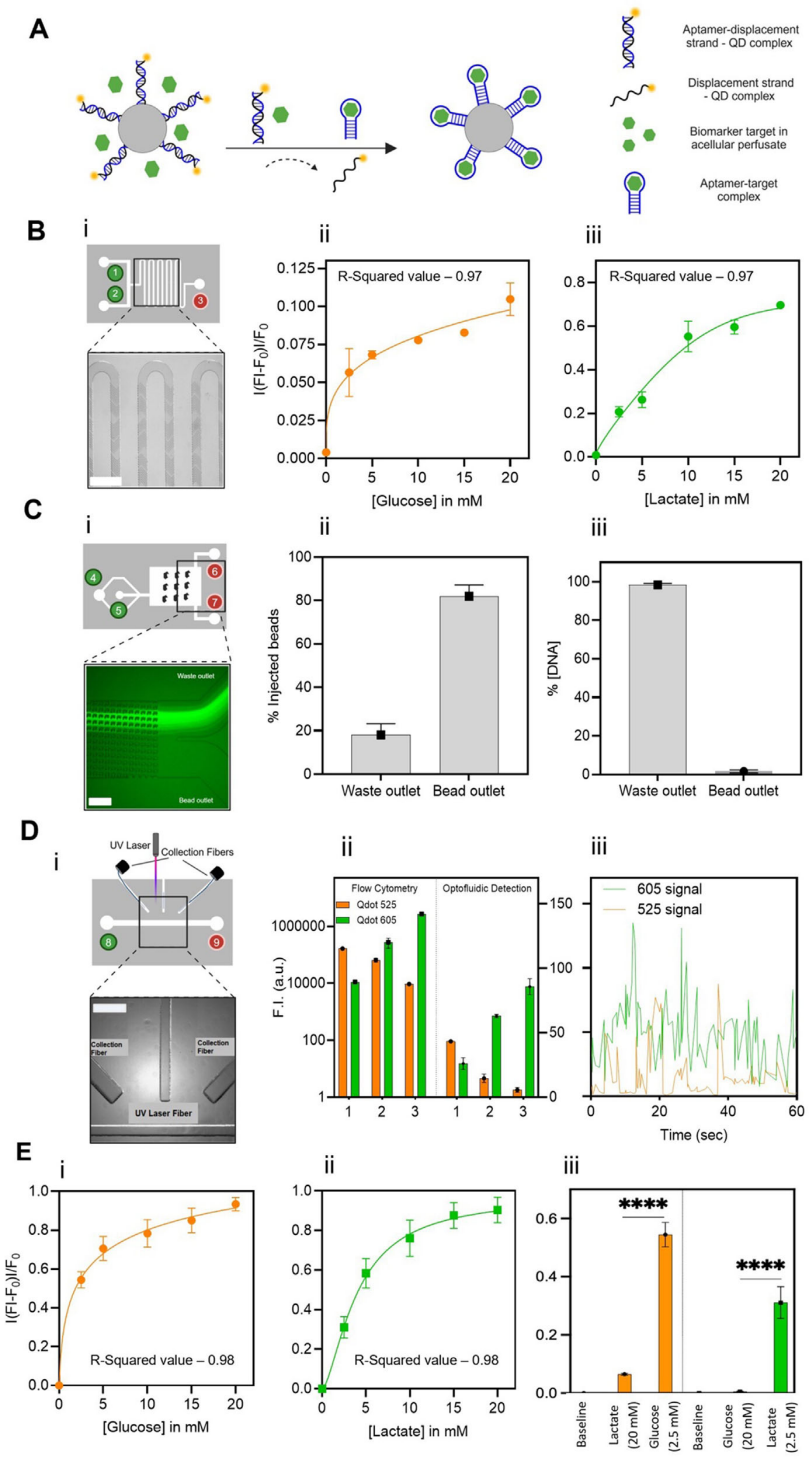
Modular microfluidic device validation. (A) Schematic depicting bead‐based, quantum dot‐mediated aptamer assay for target capture and detection. (B) Mixing module validation; i. shows the mixing module schematic and its optical image with the herringbone structure, scale bar = 1000 µm. ii., iii. Individual calibration curves of glucose and lactate were performed in the mixing module, and fluorescence intensity was measured via flow cytometry. FI is the measured fluorescence intensity of each target concentration, and F_0_ is the measured fluorescence of the blank perfusate sample. (C) Depletion module validation; i. shows the schematic of the depletion module and a fluorescence microscopy image showing fluorescent DNA being washed into the waste outlet, scale bar = 1000 µm. ii., iii. Depletion module performance through measurements of bead recovery, defined as the % injected beads recovered at the bead outlet vs. the waste outlet (ii), and depletion, defined as the concentration of unbound reagents at the waste outlet compared to the bead outlet (iii). (D) Detection module validation; i. shows the schematic and magnified image of the detection module, scale bar = 1000 µm. ii. Optofluidic and flow cytometry measurements of signals from combinations of beads functionalized with varying levels of quantum dots. Samples include #1: 100 nM quantum dot‐525 and 25 nM quantum dot‐605; #2: 50 nM quantum dot‐525 and 50 nM quantum dot‐605; #3: 25 nM quantum dot‐525 and 100 nM quantum dot‐605. iii. Unprocessed signal peaks obtained for sample #2. (E) MetaboSense validation; Glucose (i) and lactate (ii) calibration curves generated on the integrated MetaboSense platform. FI is the measured area under the peak and normalized to the fluorescence intensity of the blank solution (F_0_). iii. Assay specificity validation, where the normalized absolute value of signal gain of the highest non‐target analyte concentration (20 mM) was compared to the lowest target analyte concentration (2.5 mM) and zero analyte condition; orange shows specificity experiments against glucose and green shows against lactate. Data show mean and SD values for three replicates or devices, and comparisons are done via One‐way ANOVA with Tukey's correction. GP: < 0.0001(^****^).

MetaboSense uses a bead‐based assay and aptamer probes‐displacement strategy for detection of glucose and lactate (Figure 2A). Briefly, we hybridized glucose or lactate aptamers with a DNA competitor strand that was conjugated to quantum dots as the fluorescence molecule and coupled these complexes to polystyrene microbeads. In the absence of target, the aptamer–DNA competitor complex produces a fluorescence signal due to the presence of quantum dots conjugated to the DNA competitor. When the aptamer binds to the target, the DNA competitor strand‐quantum dot dissociates, thus the fluorescence signal decreases. The use of quantum dots particularly improved the multiplexing capability of the platform due to their unique optical properties whereby they can be excited by a single UV excitation laser and emit different discrete emission spectra. Quantum dot‐525 was designated for glucose, and quantum dot‐605 was designated for lactate.

We initially validated the performance of the bead‐based quantum‐mediated aptamer (BQA) assay in buffer solution. After functionalizing microbeads with the aptamer–DNA competitor complex, the beads were incubated with different concentrations of glucose (Figure S1‐i) or lactate (Figure S1‐ii). The fluorescence signals collected using flow cytometry show that by increasing the target concentration, the fluorescence signal decreases.

The MetaboSense platform integrates three modules to achieve continuous, real‐time monitoring (Figure 1C): (1) a mixing module, which combines molecular probes for analyte detection with the perfusate sample; (2) a depletion module, which removes the released DNA competitor‐quantum dot strand, thus minimizing the background signal; and (3) a detection module, which brings the bead‐target complex to the detection window to quantitatively measure its fluorescence signal, thus the abundance of target analyte. The design criteria and dimensions for each module were previously optimized and are shown in Figure S2 [29, 30]. We used a standard microfluidic device fabrication protocol with glass substrates and polydimethylsiloxane to build the MetaboSense device. Before fabricating the full integrated device, we first optimized the individual mixing, depletion, and detection modules. The mixing module (Figure 2B‐i) achieves rapid and continuous mixing of reagents using serpentine channels with an optimized length and incorporates herringbone structures inside the channel to create chaotic mixing. This dramatically enhances the rate of molecular diffusion and reduces the required incubation time to less than 1 min. Previously, we studied the kinetics of the bead‐based assay inside the mixing module [29, 31]. To examine the efficiency of the mixing module to form the bead‐target complex, a perfusate sample was spiked with different concentrations of glucose or lactate and injected through the sample inlet of the mixing module while the beads functionalized with glucose‐ or lactate‐specific aptamer probes and DNA competitor strands were injected through the reagent inlet of the mixing module. The reagent solution and spiked perfusate were injected at a previously optimized flow rate of 15 µL/min [29, 30]. It is important to note that the MetaboSense approach would require less than 5 mL of perfusate during a typical EVLP and thus not consume too much solution that would preclude the ease of clinical adoption. The formed BQA complexes were then collected and analyzed using flow cytometry. Fluorescence intensity of the bead–target complexes (FI) was background‐corrected by subtracting the fluorescence intensity measured from functionalized beads incubated with perfusate lacking spiked target (F_0_; blank). The resulting value was then normalized to F_0_ and reported as signal gain, which was plotted as a function of target analyte concentration (Figure 2B‐ii, iii). For clarity, we present the absolute signal change, demonstrating an increase in signal gain with increasing target concentrations. R‐squared values of 0.97 were obtained for both targets (Figure 2B‐ii, iii).

The depletion module (Figure 2C‐i) was designed based on the principle of deterministic lateral displacement and uses specific arrangements of microposts to separate particles passively based on their size. Within the microchannel, the microposts are positioned in a distinct geometric position that determines a cut‐off value to separate particles based on their size. Particles larger than the critical diameter are displaced and directed toward the bead outlet, while particles smaller than the critical diameter remain centered within the first streamline and guided toward the waste outlet. The depletion device of MetaboSense was introduced and optimized in our previous studies [29, 31] and has a theoretical critical diameter of 14 µm, meaning that particles smaller than 14 µm, which include released DNA‐competitor‐quantum dot strands, move toward the waste outlet. The BQA‐target complex, which is larger than 14 µm and has a diameter of 15 µm, is displaced toward the bead outlet. Thus, a separation of assay beads and undesired components can be achieved. Figure S2 shows the details of the depletion device, including all design criteria and sections. Figure 2C‐i shows the flow of a solution spiked with fluorescently tagged DNA, mimicking the released DNA competitor strand, being directed toward the waste outlet. Minimal fluorescence signal was observed at the bead outlet, where only beads are collected, effectively minimizing background signal in the subsequent detection module.

We next assessed the separation efficiency, attributed to the collection of beads in the bead outlet and elimination of the released DNA into the waste outlet. Buffer samples were spiked with 15 µm beads or fluorescently tagged DNA and injected through the sample inlet of the depletion module at a 15 µL/min flow rate, which corresponds with the flow rate at the outlet of the mixing module while a sheath flow solution is injected at a previously optimized flow rate of 75 µL/min. The solutions were then collected from the bead outlet and waste outlet and analyzed using flow cytometry or a plate reader for calculating bead recovery or DNA depletion efficiency, respectively (Figure 2C‐ii, iii). A bead recovery of 80% (i.e., 80% of injected beads were successfully recovered from the bead outlet) and a DNA depletion efficiency of 97% (i.e., 97% of released DNA competitor strand can be washed away) were achieved. These results verify that the depletion module can collect a large number of beads for the real‐time monitoring of target analyte while effectively depleting the released DNA competitor strand, thus minimizing the signal background.

The use of quantum dots and a specially designed optofluidic system enables multiplexed detection of glucose and lactate. The detection module deploys a microfluidic channel with three grooves – one for a 405 nm UV excitation laser and two grooves for collection fibers, one for each of the quantum dots (525 or 605) used in our assays. The collected fluorescence signals are passed through bandpass filters and photodetectors, then amplified by low‐noise current preamplifiers, allowing real‐time visualization of the signal peaks (Figure 2D‐i). The detection module was validated by comparing signals from different combinations of quantum dot–conjugated‐beads measured using the optofluidic system with those obtained from conventional flow cytometry. As shown in Figure 2D‐ii, distinct profiles were obtained for each quantum dot using the optofluidic setup at varying levels of brightness corresponding to quantum dot concentrations. We then injected a sample spiked with two populations of beads, each conjugated with one type of quantum dot used in the assay, and observed distinct profiles collected from each channel, determining that the detection module enables multiplexed measurement (Figure 2D‐iii)

Having validated the performance of the mixing module in forming glucose‐BQA and lactate‐BQA, the separation efficiency of the depletion module, and the multiplexed capability of the detection module, we then tested the individual assays for glucose and lactate on the integrated microfluidic platform. To create the integrated device, the outlet of the mixing module was connected to the sample inlet of the depletion module, and the bead outlet of the depletion module was connected to the inlet of the detection module (Figure 1C). Perfusate solutions were spiked with different concentrations (0–20 mM) of either glucose or lactate and injected through the sample inlet of the mixing module. Within the MetaboSense system, assay incubation occurs in approximately 30 s in the mixing module, followed by a 30‐s washing step and real‐time measurement in the detection module. Fluorescence signal from each bead passing through the detection channel was visualized as a peak, and area under the peak (FI) was calculated using an automated algorithm previously optimized [29, 32]. Absolute values of the normalized loss of signal upon target binding were reported as a signal gain and plotted against increasing concentrations of target analyte for both targets (Figure 2E‐i, ii). R‐squared values of 0.98 were measured for both target calibration curves, indicating a strong correlation between the signal gain measured on MetaboSense and increasing concentrations of the target analytes.

Next, assay selectivity toward the respective targets was examined. In these experiments, microbeads functionalized with target‐specific aptamer‐displacement complexes were incubated with high concentrations (20 mM) of non‐target analytes (e.g., beads functionalized with glucose‐specific aptamer‐displacement strands incubated with 20 mM lactate). The normalized signal gain was significantly different from the signal obtained from the lowest concentration of the target analyte (2.5 mM), confirming assay specificity for both glucose and lactate within the integrated MetaboSense platform (Figure 2E‐iii). To study the effect of aptamer probes on assay specificity, we incubated quantum dot‐competitor strands lacking aptamers with the maximum analyte concentration (20 mM) and compared the signal change against no‐analyte controls. As shown in Figure S3, no significant difference was observed between blank and 20 mM samples in the absence of aptamer probes, confirming that the signal generation is strictly aptamer‐dependent. It is worth noting that all in vitro experiments were performed in perfusate with a composition that is used in a clinical EVLP system [4].

### 2.3 Continuous and Multiplexed Monitoring of Glucose and Lactate in Spiked Perfusate Samples

**FIGURE 3:**
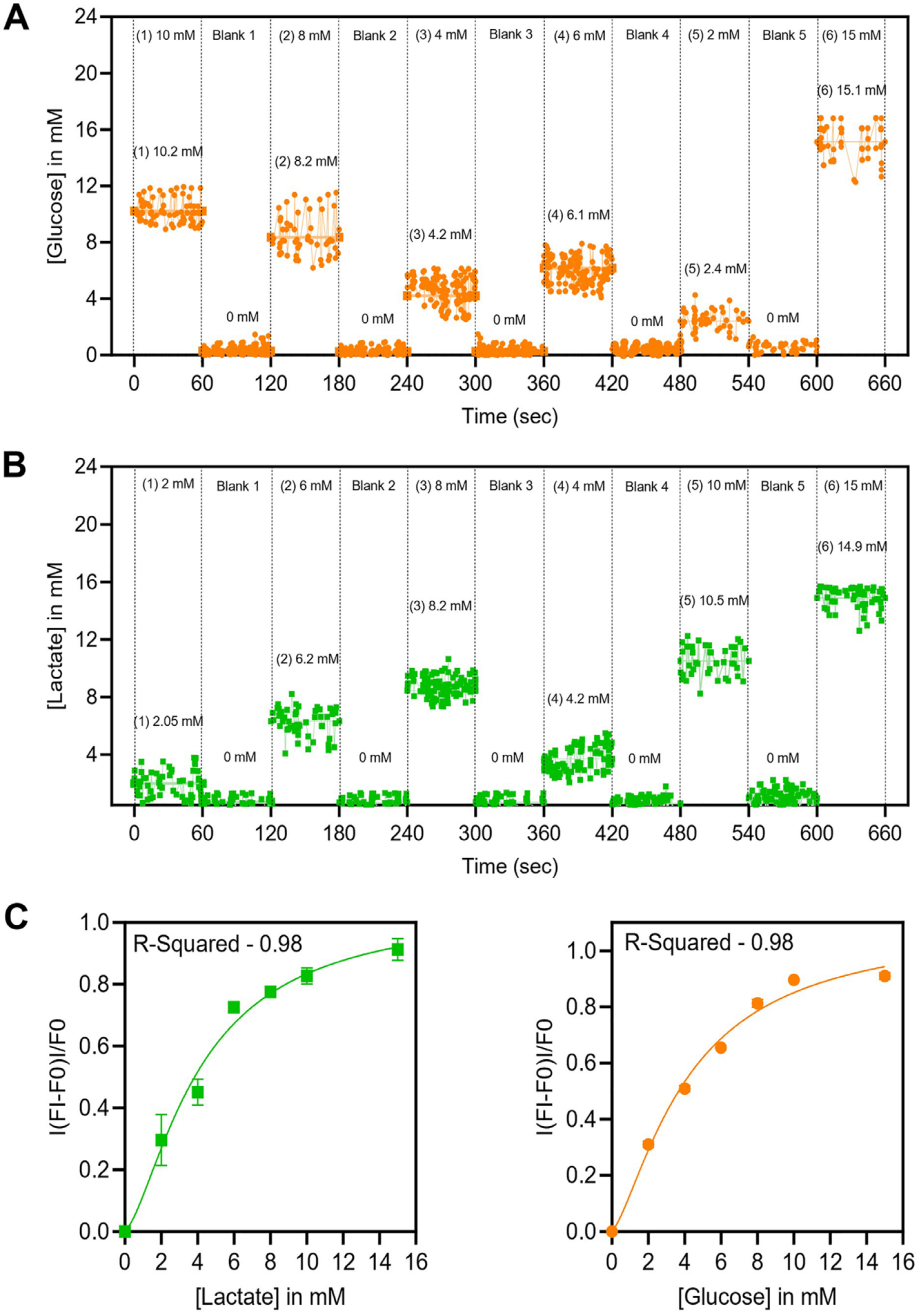
Continuous and multiplexed measurement of glucose and lactate in spiked perfusate samples. MetaboSense measurements of glucose (A, orange dot) and lactate (B, green dot) in perfusate samples relative to the spiked concentrations (line) over the course of 11 min (660 s). Calibration curves correlating the signal gain to the glucose (C‐i) and lactate (C‐ii) concentrations. Calibration curves were fitted using a sigmoidal 4PL nonlinear regression model. The experiments to derive the standard curves were repeated three times, and the error bars show the SD among three device replicates. A scan was counted as a successful measurement if at least 10 peaks/beads were observed.

Having successfully validated the performance of all individual microfluidic modules and the integrated platform to detect targets individually, we next assessed the performance of the MetaboSense platform to measure both targets in a continuous and multiplexed manner. To do this, four different sample pairs of glucose and lactate were spiked into perfusate, injected into the integrated device, and signals for both targets were measured simultaneously and continuously. Each sample pair condition was chosen to resemble the physiological concentrations of glucose and lactate during lung perfusion: 10 mM glucose and 2 mM lactate (sample pair 1), 8 mM glucose and 6 mM lactate (sample pair 2), 4 mM glucose and 8 mM lactate (sample pair 3), and 6 mM glucose and 4 mM lactate (sample pair 4). Between each sample pair, an unspiked perfusate sample was injected to wash out the modules (Figure 3A,B). In these graphs, each dot represents the concentration measurement collected from one assay bead‐target complex passing through the detection channel. We used these data to construct calibration curves that correlate the signal gain with the glucose or lactate concentration in the perfusate sample (Figure 3C‐i, ii). The limit of detection (LOD) for glucose and lactate in the continuous, multiplexed assay was 0.74 and 1.19 mM, respectively, while the limits of quantification (LOQ) were 2.5 and 3.97 mM, respectively (refer to Section 5 and Table S1 for LOD and LOQ calculations).

It is worth noting that the MetaboSense platform operates by continuously injecting the reagent solution, which consists of microbeads functionalized with the aptamer‐displacement strand complex, and bead‐based sensors are discarded following optofluidic measurement. Because the system utilizes fresh beads for each measurement, sensor regeneration is not required. In the validation experiments presented in Figure 3, different spiked perfusate samples were introduced via the sample inlet of the micromixer module while fresh beads were injected via the reagent inlet.

To evaluate the platform's performance, a continuous 2–h validation experiment was conducted using a constant lactate concentration of 10 mM (Figure S4). During these experiments, we performed device replacements at 30 and 75 min to mitigate signal overshoot caused by bead aggregation within the depletion module. Crucially, device replacement is a rapid (< 5‐min) procedure that results in minimal data loss.

### 2.4 Clinical Validation of MetaboSense Platform

After extensively validating all components of the platform, we proceeded to assess its performance using clinical samples. The MetaboSense system was employed to measure glucose and lactate levels in eleven EVLP cases, with samples collected at hourly intervals (donor demographic data is summarized in Table S2). Calibration curves (shown in Figure 3) were used to determine the concentrations of glucose and lactate from MetaboSense measurements. These results were then compared to gold‐standard arterial blood gas (ABG) measurements obtained on‐site during the EVLP procedures.

**FIGURE 4:**
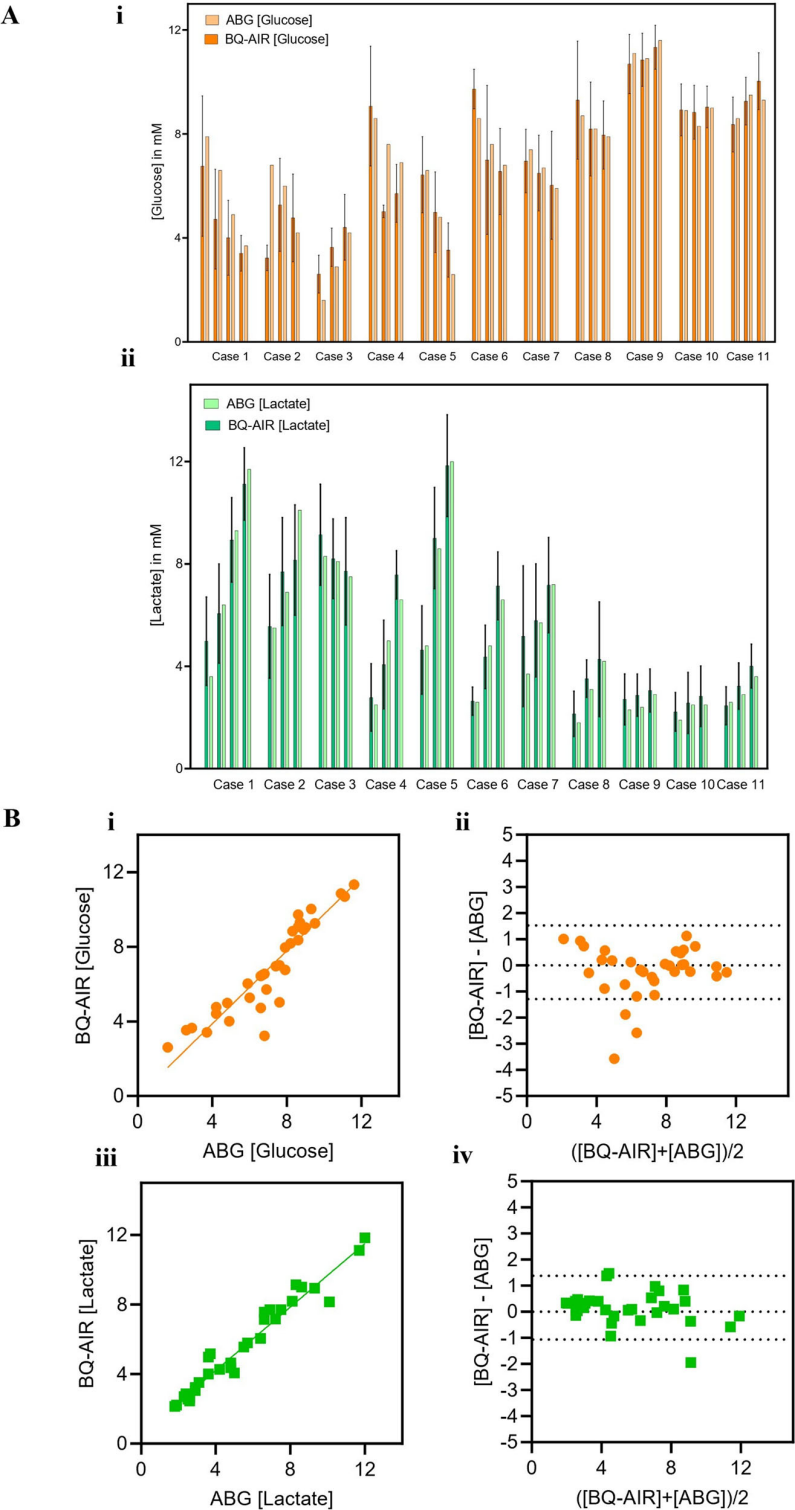
Clinical validation of MetaboSense. (A) Glucose (i) and lactate (ii) levels measured by MetaboSense and ABG in 11 EVLP cases. Columns represent individual concentration values for each time point in each EVLP case measured using ABG, and the values for each sample measured using MetaboSense. ABG data reflects a single concentration measurement, and data from the MetaboSense shows the mean and SD of a minimum of 18 scans taken. (B) Glucose (i) and lactate (iii) linear correlation of MetaboSense measurements against ABG. Glucose (ii) and lactate (iv) Bland‐Altman analysis showing [measurements from MetaboSense] – [measurements from ABG] on the x‐axis against [average of individual measurements from both methods] on the y‐axis. The majority of data points fall within the limits of agreement, with three (glucose) and one (lactate) data points falling beyond this range.

The measurements obtained from MetaboSense were plotted against clinically measured glucose and lactate concentrations (Figure 4A‐i, ii). To evaluate agreement between the two methods, we performed both Bland–Altman analysis and linear correlation (Figure 4B‐i–iv). The correlation coefficients for glucose and lactate were 0.92 and 0.97, respectively, indicating a strong linear relationship and measurement accuracy in clinically relevant ranges (Figure 4B‐i, iii). The Bland–Altman analysis further confirmed the consistency between MetaboSense and ABG measurements by plotting the mean of each pair of measurements against their difference. For both glucose and lactate, the data points remained within the accepted range of zero bias and standard deviation of error (Figure 4B‐ii, iv), supporting the reliability of the MetaboSense platform. A few data points were present outside the limits of agreement (*n* = 3 for glucose; *n* = 1 for lactate). These deviations can be attributed to inherent discrepancies between the amperometric biosensing used in reference ABG measurements and the optical MetaboSense readings. Although the clinical samples were not measured continuously, MetaboSense enabled real‐time, multiplexed detection. These results highlight the potential of MetaboSense for integration into critical care and transplant settings.

### 2.5 MetaboSense Integration With EVLP System for In‐Line Monitoring

**FIGURE 5:**
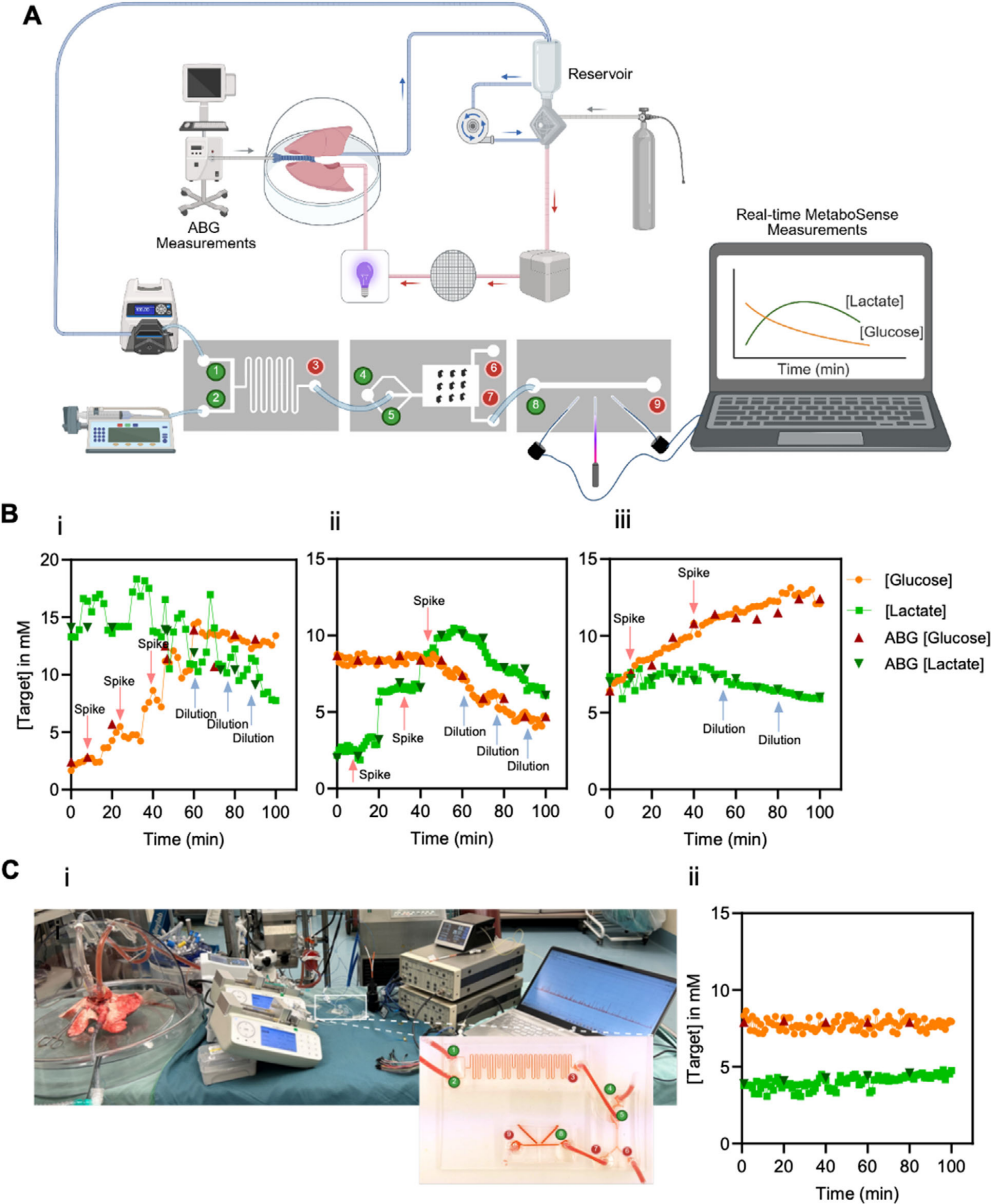
MetaboSense integration with EVLP for in‐line monitoring. (A) Schematic of MetaboSense system integrated within an EVLP circuit with a porcine lung. (B) Testing MetaboSense with pre‐set concentrations of glucose and lactate in EVLP perfusate to mimic different metabolic activities in the lung. Target concentrations were continuously monitored by MetaboSense and correlated with ABG measurements taken approximately every 10 min. (i) The first spiked experiment showing increasing levels of glucose and declining levels of lactate in an EVLP circuit, (ii) the second spiked experiment showing declining levels of glucose and increasing levels of lactate in an EVLP circuit, and (iii) the final spiked experiment with median glucose and lactate levels. Concentrations of each target measured by MetaboSense were determined by interpolation from the corresponding calibration curves. (C) (i) Image of EVLP circuit with a porcine lung assessed along with in‐line MetaboSense platform connected to the perfusate reservoir. Magnified inset shows MetaboSense's microfluidic components. (ii) A single healthy pig lung was assessed with the standard Toronto EVLP protocol. The glucose and lactate levels remained stable, indicating normal lung function. MetaboSense recorded measurements every minute over a 100‐min period. Given the consistency of metabolite levels, ABG sampling was conducted every 20 min for comparison. Two or three MetaboSense devices were used per in‐line experiment, and ABG data reflect a single measurement.

To further extend the clinical validation of the MetaboSense, glucose and lactate measurements were performed in‐line within an EVLP circuit after removal of porcine lungs as well as an EVLP circuit with porcine lungs being perfused (Figure 5A). To do this, the MetaboSense was connected to the EVLP circuit through the sampling port using a lure lock and peristaltic pump tubing, which directed the perfusate into the device. Additionally, the EVLP sampling port was used to draw perfusate from left atrial and pulmonary arterial circulation for on‐site ABG measurements for cross‐validation. Initially, MetaboSense bead recovery and depletion were assessed in‐line and determined to be 90% and 95%, respectively (Figure S5 i, ii). Next, continuous and multiplexed glucose and lactate measurements were performed within an EVLP circuit following the removal of porcine lungs. Three distinct perfusate conditions were externally spiked to replicate clinically relevant scenarios. These conditions were assessed using the MetaboSense system (Figure 5B). In the first scenario (Figure 5B‐i), the EVLP perfusate reservoir initially contained high lactate and low glucose levels following lung removal, mimicking the metabolic conditions associated with lung injury. Glucose levels were subsequently increased at regular intervals, while lactate levels were gradually reduced to simulate gradual recovery of lung metabolism during EVLP. Glucose and lactate concentrations were continuously monitored at 1‐min intervals using MetaboSense and validated against ABG measurements taken approximately every 10 min. In the second scenario (Figure 5B‐ii), the EVLP circuit contained high initial glucose and low lactate levels, mimicking a lung with relatively normal metabolism. Lactate was subsequently spiked, and glucose was diluted to simulate metabolic shifts toward deterioration during EVLP. These changes were continuously monitored using MetaboSense. In the third scenario (Figure 5B‐iii), perfusate with moderate initial glucose and lactate levels was tested. Over time, glucose levels were gradually increased while lactate levels were slightly decreased, demonstrating the system's ability to detect metabolic fluctuations of donor lungs with relatively stable metabolism during EVLP.

Following validation of MetaboSense in an EVLP circuit after the removal of porcine lungs and with spiked/diluted glucose/lactate levels in perfusate, we performed EVLP with a healthy porcine lung undergoing perfusion on EVLP (Figure 5C‐i). Notably, glucose and lactate were not externally spiked into the reservoir; instead, their endogenous physiological levels were continuously monitored by MetaboSense and compared with ABG measurements. MetaboSense collected data at 1‐min intervals throughout the entire perfusion period (∼100 min), while ABG samples were taken every 20 min. The lung exhibited minimal fluctuations in glucose and lactate levels during this time, indicating stable metabolic function and overall robust health (Figure 5C‐ii). Correlations between ABG measurements and interpolated MetaboSense readings for both glucose and lactate across all in‐line measurements are shown in Figure S5‐iii, iv. The R‐squared values of 0.97 for glucose and 0.98 for lactate demonstrate the strong alignment between MetaboSense and the gold‐standard ABG analyzer.

## 3 Discussion

The present study discusses the development and validation of MetaboSense, a modular microfluidic platform for continuous, real‐time measurement of glucose and lactate during EVLP. We demonstrate successful integration of multiplexed aptamer‐based assays into a device capable of in‐line perfusate monitoring, with strong agreement against gold‐standard blood gas analyzers across a range of test settings. These include clinical EVLP perfusate samples, flowing EVLP circuits, and in‐line perfusion of porcine lung grafts, reflecting the platform's consistency across diverse and relevant testing environments. Unlike prior studies focused on the prognostic role of glucose and lactate in EVLP, our work focuses on enabling the technical realization of continuous biomarker capture during EVLP. By showing that accurate, high‐resolution measurements can be achieved using a low‐volume assay system, this work establishes a foundation for future integration of automated biochemical monitoring into clinical perfusion workflows that enhance data capture while minimizing procedural burden for EVLP teams. To extend operational duration for multi‐hour perfusion applications, future iterations of the platform will incorporate integrated filtration stages upstream of the depletion module to effectively prevent bead aggregation.

Upgrading biochemical monitoring from intermittent to continuous formats aligns with broader efforts to enhance the temporal resolution of physiological and biological data for health and disease modeling. In critical care, high‐frequency ventilator waveforms have been used to train predictive models for respiratory deterioration, acute respiratory distress syndrome (ARDS) detection, and extubation readiness in infants [33, 34, 35, 36, 37]. Other continuous data streams — including electrocardiogram and oxygen saturation waveforms — have improved clinical outcomes, with continuous monitoring linked to reduced ICU transfers, mortality, and hospital stays by enabling earlier detection of deterioration [38, 39]. Single‐modality signals such as electrocardiogram waveforms have supported diverse modeling applications in health and disease [40, 41, 42], while multimodal continuous vital sign data have advanced diagnostic and prognostic models of complex syndromes such as sepsis [43, 44, 45]. Within EVLP, ventilator pressure and flow traces provide continuous physiologic information, yet most biological and biochemical parameters remain limited to intermittent measurement — constraining the development of richer, multimodal models for predicting EVLP outcomes. Expanding continuous sensing to these domains represents a key opportunity to improve EVLP decision‐making to optimize organ utilization and transplant recipient outcomes.

MetaboSense was validated using multiple measurements obtained from eleven clinical EVLP cases for a total of 34 independent timepoint samples. This is the largest known cohort of its kind and demonstrates the clinical feasibility of the MetaboSense platform. Future large‐scale validation studies will establish the broader clinical utility of MetaboSense. Beyond graft assessment, continuous real‐time metabolite monitoring may enable closed‐loop control of EVLP, whereby perfusion parameters are dynamically adjusted in response to the metabolic state of the organ to optimize organ support and improve transplant outcomes. Additionally, MetaboSense's modular design presents a promising opportunity to extend real‐time monitoring during EVLP to new biomarker classes. Chief among these are circulating pro‐ and anti‐inflammatory cytokines such as IL‐6, IL‐8, IL‐10, and IL‐1β, which have been demonstrated to play central roles in the pathophysiology of lung injury and graft viability, as well as serve as key predictors of EVLP and recipient outcomes [3, 6, 7, 46, 47, 48]. At present, cytokine quantification relies on laboratory immunoassays performed on intermittently collected perfusate samples with turnaround times that exceed the window of clinical EVLP, limiting their intra‐procedural utility. In principle, MetaboSense's bead‐based architecture could be extended to cytokine measurement by replacing the current aptamer–displacement probes with antibody‐based immunoassays, while retaining the same mixing, depletion, and detection modules for real‐time detection. This modified platform could enable real‐time continuous characterization of the inflammatory milieu during perfusion, offering both mechanistic insights into lung health and actionable clinical guidance during EVLP.

Beyond the lung, continuous monitoring of glucose and lactate has direct relevance to other ex vivo organ perfusion platforms, including those for liver, kidney, heart, and pancreas transplantation, where glucose and lactate dynamics are also critical determinants of organ quality [17, 18, 19, 20]. The same modular platform could thus serve as a versatile backbone for multiplexed biochemical monitoring across transplantation domains, expanding its impact beyond EVLP and accelerating the adoption of continuous sensing in clinical practice.

## 4 Conclusion

MetaboSense is the first microfluidic platform to enable real‐time, continuous, and multiplexed measurement of glucose and lactate during EVLP. By integrating a bead‐based aptamer assay with modular microfluidic design, the system delivers accurate, high‐resolution biochemical monitoring with strong agreement to clinical standards. This work establishes a technical foundation for automated, in‐line metabolic assessment during EVLP, reducing reliance on intermittent sampling and manual analysis. With its modularity, the platform is well‐positioned for future adaptation to additional biomarkers, supporting broader efforts to integrate continuous biochemical sensing into clinical organ perfusion.

## 5 Material and Methods

### 5.1 Materials

All chemicals for preparation of buffers and reagents were purchased from Sigma–Aldrich, Qdot conjugation ITK carboxyl kits were purchased from Thermofisher, aptamers were purchased from Integrated DNA Technologies., Streptavidin‐coated 15 µm polystyrene microspheres were purchased from Bangs Laboratories Inc. Streptavidin coated 10 µm magnetic beads were purchased from Spherotech Inc. Carboxylate‐coated 15 µm beads for quantum dot functionalized beads used in optofluidic characterization were purchased from Spherotech Inc. Streptavidin‐Qdot 605 and 525 were purchased from Thermofisher. Polydimethylsiloxane (PDMS) was purchased from Ellsworth Adhesives (Sylgard 184 kit). All optical components were purchased from Thorlabs, Newport, and Stanford Research Systems.

Aptamer and displacement strand sequences for lactate for initial plate reader and flow cytometry validations are as follows:

/5BiosG/i6FAMK/TTTTTTTCTCTCGACGACGAGTAGCGCGTATGAATGCTTTTCTATGGAGTCGTC and CGTCGTCGAGAG/3IABkFQ/.

Aptamer and displacement strand sequences for glucose for initial plate reader and flow cytometry validations are as follows:

/5BiosG/iCy3/CTCTCGGGACGACCGTGTGTGTTGCTCTGTAACAGTGTCCATTGTCGTCCC and GGTCGTCCCGAGAG/3DAb/.

Aptamer and displacement strand sequences for lactate signal‐off qdot assays are as follows:

/5BiosG/TTTTTTTCTCTCGACGACGAGTAGCGCGTATGAATGCTTTTCTATGGAGTCGTC and CGTCGTCGAGAG/3AmMO/.

Aptamer and displacement strand sequences for glucose signal‐off qdot assays are as follows:

/5BiosG/CTCTCGGGACGACCGTGTGTGTTGCTCTGTAACAGTGTCCATTGTCGTCCC and GGTCGTCCCGAGAG/3AmMO.

Lactate SELEX buffer recipe is as follows: 500 mM NaCl, 50 mM HEPES buffer, 10 mM MgCl_2_, 10 mM KCl. The glucose SELEX buffer recipe is as follows: 1 M NaCl, 20 mM HEPES buffer, 20 mM MgCl_2_, 20 mM KCl.

### 5.2 Preparation of Quantum Dot Functionalized Beads

Carboxylate‐functionalized magnetic beads (15 µm) purchased from Spherotech Inc. were functionalized with Streptavidin‐Qdots to facilitate optofluidic characterization. The supplier's protocol was used for this functionalization. Briefly, carboxylate‐functionalized beads were treated with 1‐ethyl‐3‐(3‐dimethylaminopropyl) carbodiimide (EDC) and N‐hydroxysuccinimide (NHS) in 2‐(N‐morpholino) ethane sulfonic acid (MES) pH 5.2 buffer for 30 min at room temperature to activate the carboxyl groups on the surface. Beads were then incubated with Streptavidin‐conjugated Qdot 605 or 525 in three different concentrations – 100, 50 and 25 nM – to generate six groups of beads. These were incubated at room temperature for 4 h, before being washed with 1X PBST (1X PBS + 0.05% Tween‐20) thrice and resuspended in 1X PBSBT (1X PBS + 0.01% Tween‐20 + 0.05% BSA).

### 5.3 Benchtop Bead‐Based Quantum Dot Mediated Assays

Amino‐modified displacement strands were conjugated with carboxyl‐modified quantum dots as per the protocol outlined in Thermofisher ITK carboxyl quantum dot kits. Conjugation efficiency was determined by the molar ratio of displacement strand to quantum dot in the purified conjugate. Following carboxyl‐Qdot coupling per the Thermo Fisher protocol, unreacted strand was removed by centrifugal ultrafiltration (Amicon Ultra‐0.5, 100 kDa molecular weight cut‐off, Ultracel regenerated‐cellulose membrane; MilliporeSigma UFC5100) with reverse‐spin retentate recovery, retaining the Qdot–DNA conjugate while free ∼4 kDa (3.9 kDa lactate and 4.5 kDa glucose displacement sequence) strands partitioned into the filtrate. The retentate was then interrogated by UV–vis spectrophotometry at a 1 cm pathlength: oligonucleotide concentration was derived from A_260_ and Qdot concentration from absorbance at the characteristic excitation wavelengths (350/405/488/532 nm), each resolved through the Beer–Lambert relationship (C = A/εl) using the respective molar extinction coefficients (Qdot 525 and Qdot 605 per Thermo Fisher MP19020; displacement strand ε computed from IDT). Conjugation efficiency, expressed as number of DNA strands per Qdot, was 83.4 ± 0.1 (DNA:Qdot) for the lactate‐displacement strand–Qdot 605 construct and 282 ± 14 (DNA:Qdot) for the glucose‐displacement strand–Qdot 525 construct (mean ± SD, *n* = 5 replicates) when taking the ratio of the DNA concentration (A_260_) to the Qdot 605 concentration derived from absorbance at 405 nm as a representative excitation wavelength.

Displacement strands were then hybridized with their respective aptamers in optimized molar ratios (4:1 aptamer: displacement strand for lactate, and 1:1 aptamer: displacement strand for glucose) with their respective SELEX buffer for 20 min at room temperature, before incubation with 15 µm streptavidin coated polystyrene microspheres for 30 min at room temperature. Beads were washed and resuspended in SELEX buffer prior to target incubation. 2E4 beads were incubated with perfusate spiked with increasing concentrations of target analytes individually at room temperature for 40 min, followed by final washes and fluorescence emission measurement using flow cytometry (CytoFlex, Beckman Coulter).

### 5.4 Number of Quantum Dots per Bead

To estimate the number of quantum dots (Qdots) per bead, we first determined the number of aptamer probes immobilized on the bead surface. This was estimated based on the manufacturer's biotin‐binding capacity (2.46 nmol/mg) for streptavidin‐coated polystyrene microspheres (15 µm diameter; Bangs Laboratories, catalog # CP01008). Given a polystyrene density of 1.05 g/cm^3^ and a mean bead diameter of 15 µm, we calculated ∼5.39 × 10^5^ microspheres per milligram. Assuming a 1:1 biotin‐to‐streptavidin stoichiometry, this corresponds to a theoretical maximum of ∼2.7 × 10^9^ biotinylated aptamer molecules immobilized per bead. Applying a 1:1 and 4:1 aptamer‐to‐displacement strand ratio for the glucose and lactate assays, respectively, yields approximately 2.7 × 10^9^ glucose displacement strands and 0.675 × 10^9^ lactate displacement strands per bead. Based on our conjugation efficiencies (282 DNA:Qdot for glucose; 83.4 DNA:Qdot for lactate), we estimate approximately 9.6 × 10^6^ Qdots for glucose beads and 8.1 × 10^6^ Qdots for lactate beads.

### 5.5 MetaboSense Device Fabrication

First, the microchannels for the mixer and depletion modules were designed on AutoCAD and KLayout software. The masks needed for the two‐layer photolithography of the mixer and the depletion module were printed (CAD/Art Services). An SU‐8 3050 (MicroChem) layer of 45 µm was coated on a Silicon wafer (University Wafer) for the first layer, which was the main channel of the mixer. Then, the same height of SU‐8 3050 was coated on the developed first layer to pattern the herringbone structures. Both steps were fabricated using a mask aligner with UV light (MA6, SUSS MicroTec). The process for the master mold of the depletion module followed a one‐layer fabrication protocol. The channel height for the devices (also including the micropost) was 50 µm, and it was carried out using UV Direct Write photolithography (MLA 150, Heidelberg Instruments). The microfluidic devices were fabricated following a standard polydimethylsiloxane (PDMS)‐based method. The cast PDMS on the master molds was peeled off and bonded to glass slides (Electron Microscopy Sciences) using a plasma etcher (Tergeo Plasma Cleaner, Pie Scientific) with the following settings: 15 W, 30 s, 10 sccm, air. Prior to the experiment, all the devices were degassed with a non‐ionic surfactant 1% Pluronic F108 solution.

### 5.6 Optofluidic Measurement

The optofluidic setup was used to detect and convert the signals from our two target quantum dots – 605 and 525 for lactate and glucose, respectively. A universal excitation 405 nm laser (Model: LP405‐MF300, Thorlabs) with 300 mW optical power was used to excite beads flowing through the sample channel in the detection module. A 260 µm (Model: M137L02, NA: 0.22, Thorlabs) optical fiber was inserted within the excitation groove in the microfluidic chip to facilitate precisely guiding the laser light, minimizing energy loss, reducing light divergence, and allowing concentrated illumination at the point of interrogation in the sample channel. Similarly, two 425 µm (Model: M118L02, NA: 0.39, Thorlabs) optical fibers were inserted into the two collection grooves of the detection module at a 45° angle on the same side as the excitation fiber, facilitating the collection of emitted fluorescence signal without direct exposure to the excitation light, minimizing the interference effect. Two bandpass filters, 610/10 nm (Model: FBH610‐10, Thorlabs) and 660/10 nm (Model: FBH530‐10, Thorlabs) were chosen to allow only the relevant fluorescence from quantum dots to pass through. The collected and filtered fluorescence light was then passed through a focusing lens (Model: LB1494‐A, Thorlabs) before being directed onto the detector's active area. Two Si‐pin power photodiodes (Model: 818‐UV/DB, Newport) were used to convert the fluorescence signal into an electrical signal. To overcome the inherent challenges of collecting an electrical signal equivalent to the weak optical signal from the beads conjugated with quantum dots, two low‐noise current preamplifiers (Model: SR570, Stanford Research Systems) were deployed, one for each quantum dot signal, to ensure that even the weakest signals from beads’ surfaces were detected and measured. The output electrical signals were processed through an oscilloscope and saved as scans of voltage peaks for each quantum dot signal from beads over time on a laptop.

### 5.7 Modular Microfluidic Device Characterization

The performance of the depletion module regarding the bead recovery and purity was evaluated using flow cytometry (NovoCyte, Agilent). For each bead recovery, the number of collected beads from the bead outlet was analyzed and compared to the number of injected microbeads at the inlet by gating and measuring the number of beads collected. The purity was assessed by flowing a solution of FITC‐conjugated‐DNA through the inlet of the depletion module, and the corresponding concentration of collected DNA was measured in the bead and waste outlets using a plate reader.

The optofluidic detection module was assessed by running three different combinations of Qdot‐functionalized beads that were prepared as described in the section above through the integrated MetaboSense device, and their signals were measured with the detection module. The final output flow from the last outlet of the detection module was collected for each bead combination and assessed using flow cytometry. The combinations were as follows: 100 nM 525 with 25 nM 605, 50 nM 525 with 50 nM 605, and 25 nM 525 with 100 nM 605. These combinations were chosen to reflect the trends in the signals of these quantum dots as each of their assigned targets’ concentrations change during EVLP.

### 5.8 On‐Chip BQA – Individual Calibration Curves, Specificity, and Multiplexed Measurement

Beads were prepared as outlined in the benchtop section but resuspended in SELEX buffer + 20% sucrose solution as a device running buffer. Perfusate samples were spiked with known concentrations of target analytes. Microfluidic devices were prepared and degassed as outlined in the device fabrication protocol. A syringe was filled with ∼600 µL of reagents (∼2E4 target capture beads) and running buffer (SELEX + 20% sucrose), and a second syringe (sample syringe) was filled with spiked perfusate corresponding to each experimental case. These were connected to a syringe pump and flown through an integrated MetaboSense device at a flow rate of 15 µL/min and a depletion module sheath flow rate of 75 µL/min. Each sample was run until enough scans had been obtained for data analysis of peak amplitude. Each scan had a duration of ∼60 s, and each sample was run for a minimum of 3 scans.

For specificity experiments, reagent syringes were maintained, but sample syringes were switched to those containing 20 mM of a non‐target analyte, and the same data collection procedure was followed. For multiplexed experiments, reagent syringes contained capture beads for both targets (∼1E4 beads each for a total of 2E4 beads) and sample syringes contained perfusate spiked with specific combinations of glucose and lactate corresponding to four different physiological cases. Data collection procedures were the same as described for individual calibration curves.

### 5.9 On‐Chip Continuous Monitoring of Glucose and Lactate in Perfusate Samples

MetaboSense devices were fabricated and degassed as outlined in the device fabrication protocol. A syringe filled with a larger volume (∼1 mL) of assay reagents (capture beads with Aptamer‐Qdot‐displacement strand complex) and another (sample syringe) filled with perfusate spiked with targets to a final volume of 300 µL were fitted into a syringe pump and flown through the degassed MetaboSense platform. Each sample syringe contained a distinct test condition. Test conditions were as follows: 10 mM glucose with 2 mM lactate, 8 mM glucose with 6 mM lactate, 4 mM glucose with 8 mM lactate, and 6 mM glucose with 4 mM lactate. Between each concentration, an unspiked perfusate sample was run through. Sample syringes were each run until enough volume was obtained to obtain signals for three to 4 min. The sample syringe was then switched out to the next consecutive sample, while keeping the reagent syringe unchanged. A flow rate of 15 µL/min was used for injecting reagents and samples into the mixer module, and a flow rate of 75 µL/min was used as the depletion module sheath flow rate. Samples were run until sufficient peaks were obtained for both targets.

### 5.10 Clinical Validation of MetaboSense Platform

Frozen perfusate samples (∼2 mL total volume) taken at hourly intervals from eleven clinical EVLP cases were used to validate the MetaboSense system in measuring glucose and lactate levels in clinical samples. These samples were evaluated initially using traditional arterial blood gas (ABG) measurement (GEM 5000 Blood Gas Analyzer) to determine glucose and lactate levels at hourly intervals. Samples were thawed immediately prior to use in validation experiments. MetaboSense devices were fabricated and degassed prior to use as described above. Assay reagents were prepared as outlined in the sections for generating benchtop bead‐based assays. Briefly, displacement strands were conjugated to their respective quantum dots following the Thermofisher ITK protocol, followed by incubation with their aptamers in optimized molar ratios, and incubation with streptavidin‐functionalized beads. These were then washed and resuspended in SELEX. 1 mL syringes were filled with 2E4 beads in total for both targets in running buffer (SELEX+ 20% sucrose). Thawed samples were run in their entirety; however, divided into three by volume, with multiplexed detection of targets and scans collected continuously until the whole sample volume was run through (∼20 scans per sample). Scans were then processed through the data analysis app described below, followed by statistical analysis to get signal gains that were then interpolated into concentrations using the calibration curves derived from the continuous, multiplexed spiked perfusate experiments.

### 5.11 Spiked In‐Line EVLP Experiments

Reagents were prepared as described as outlined in the sections for continuous glucose and lactate in vitro experiments. MetaboSense devices were fabricated and degassed as per procedures outlined in the section for device fabrication. An EVLP circuit with STEEN solution (a standard acellular EVLP perfusion solution) was initially assessed for residual glucose and lactate levels using ABG, as baseline levels. A 1 M glucose solution in STEEN solution was prepared to spike the circuit. To begin monitoring with the integrated MetaboSense system, a peristaltic pump (Cole Parmer, 78001–62) was connected to the perfusate reservoir to continuously withdraw perfusate into the mixer module of the MetaboSense device. Flow in the EVLP circuit was maintained between 1.5 and 1.6 L/min. Peristaltic pump flow was set to maintain an inlet flow rate of 15 µL/min at the mixer module of the MetaboSense device. Reagents were added to a 1 mL syringe and attached at the second mixer inlet, with a flow rate of 15 µL/min. The depletion module sheath buffer flow rate was set to 75 µL/min. Optofluidic excitation and collection fibers were inserted into their assigned grooves in the detection module, and preamplifiers were arranged along with the laser driver and oscilloscope in accordance with the schematic shown in Figure 5A. Perfusate from the reservoir was measured for 10 min by MetaboSense to measure baseline levels of glucose and lactate. Scans were collected every minute for the duration of the entire experiment. For SI Figure 4iii, iv, the first spike of 2.5 mM of glucose was added into the reservoir by approximating the total volume of perfusate in circulation in the reservoir and EVLP tubing. Subsequent spikes of glucose were determined based on the initial level of glucose and ABG readings taken during the experiment. Levels of glucose and lactate were allowed to equilibrate for a minimum of 10 min before performing a spike. At the 60‐min time point, lactate levels were brought down by removing perfusate from the circuit and adding fresh STEEN solution into the circuit at an equivalent volume. This was also allowed to equilibrate for 10 min before further removals were performed. Glucose and lactate levels were measured on‐site by ABG for collected perfusate samples, in addition to perfusate samples being collected at 5‐min intervals for later processing. In Figure 5B–i, lactate was spiked into the EVLP circuit after lung removal – this was performed using a similar method as described above for glucose, only using a 1 M lactate solution in STEEN. Lactate and glucose levels were measured continuously by MetaboSense and sampled at 5‐min intervals for ABG measurements similarly. To perform circuit dilutions, volumes of perfusate were withdrawn from the reservoir, and equivalent volumes of Milli‐Q water were added. These volumes were determined by approximating the total volume of perfusate in the reservoir and EVLP circuit tubing. All data analyses for these experiments were performed as described below.

### 5.12 In‐Line EVLP Perfusion Experiments With a Porcine Lung

Institutional Approval was obtained for the collection, storage, and analyses of the biospecimens and data used in this study (UHN REB#12‐5488‐13 and UHN REB#11‐0170‐AE). A porcine lung was connected to the EVLP circuit, following the standard Toronto EVLP protocol [4]. MetaboSense devices, reagents, a peristaltic pump, and detection components were prepared as described above. Lactate and glucose levels were measured by ABG initially to determine baseline levels. MetaboSense measurements of glucose and lactate were taken continuously, totalling 100 scans with a duration of 60 s per scan. For this experiment, the EVLP circuit was not spiked, maintaining the physiological conditions as per standard practice. ABG perfusate samples were collected at 5‐min intervals. Data analyses on all scans collected were performed as described below.

### 5.13 Data Analysis

To facilitate the automated and rapid data analysis for the discrete and continuous experiments involving the integrated MetaboSense devices and detection module, a custom app was developed that was used to extract data from optofluidic scans and convert the individual voltage peaks from beads into normalized signal gains that can be used to generate calibration curves or interpolate concentrations from pre‐generated calibration curves. The app initially extracts data from both channels of quantum dots (525 channel for glucose and 605 channel for lactate). Next, the app can smooth the signal for either QDot 605 or QDot 525 channels using the Savitzky‐Golay filter if any smoothing is necessary for scans that may be noisy from external interference. Next, a package that utilizes an iterative polynomial regression algorithm is used to estimate and remove the undesired baseline from the signals, making it possible to calculate the area under the peaks. Then, the app finds the peaks in the signals if the condition of exceeding 10% of the maximum peak amplitude is satisfied in the signal peaks and integrates the area under each peak. Finally, the average area under the peaks for each scan is obtained, which can then be analysed into a normalized signal gain readout by using absolute values of the signal loss. The developed code in the app can be found online at https://github.com/hesam‐abouali/2B‐QIRT‐ELISA, or the app can be directly used from the Streamlit web apps repository at https://2b‐qirt‐elisa.streamlit.app.

### 5.14 Statistical Analysis

All statistical analyses were conducted using GraphPad Prism 10. Each experiment was carried out in three parallel replicates. All data are presented as mean ± SD of three experimental replicates. The calibration curves were calculated using the sigmoidal 4PL nonlinear regression model. The LOD and LOQ were calculated by the formula LOD = 3 × SD/S and LOQ = 10 × SD/S, respectively, where SD is the standard deviation of the blank sample, and S is the slope of the linear range of the calibration curve [49] (Table S1). Interpolations of individual concentrations were performed using normalized absolute values of signal loss, and a 95% confidence interval from calibration curves generated for continuous, multiplexed in vitro experiments.

## Author Contributions

Conceptualization: S.S., A.T.S., M.P., M.L., M.C., S.K.; Methodology: S.S., T.B., P.K.M., R.L., H.A., G.S., G.G.; Software: S.S., P.K.M., H.A.; Formal Analysis: S.S., T.B., P.K.M., L.R.; Detection Module Development: M.F.A.F., D.B.; Investigation and Data Curation: S.S., T.B., P.K.M., L.R., G.S., G.G., I.J., H.A.; Visualization: S.S., T.B., P.K.M.; Writing – Original Draft: S.S., T.B.; Writing – Review & Editing: S.S., T.B., P.K.M., R.L., G.S., G.G., H.A, A.T.S., M.P., M.L., M.C., S.K.; Supervision: S.S., T.B., A.T.S., M.P., M.L., M.C., S.K.; Funding Acquisition: A.T.S., M.P., M.L., M.C., S.K.

## Conflicts of Interest

S.K. serves as Chief Medical Officer of Traferox Technologies and receives personal fees from Lung Bioengineering, outside the submitted work. M.C., A.T.S., and S.K. are inventors of patents related to the submitted work. M.P. is the inventor of a patent related to the submitted work. The inventors fully adhere to policies at their institutes that ensure academic integrity and management of potential conflicts of interest. The remaining authors declare no competing interests.

## Supporting information




**Supporting File**: advs77123‐sup‐0001‐SuppMat.docx.

## Data Availability

The data that support the findings of this study are available from the corresponding authors upon reasonable request.

## References

[1] J. C. Yeung , R. Zamel , W. Klement , et al., “Towards Donor Lung Recovery—Gene Expression Changes during Ex Vivo Lung Perfusion of human Lungs,” American Journal of Transplantation 18, no. 6 (2018): 1518–1526, 10.1111/ajt.14700. 29446226

[2] J. M. Garijo and A. Roscoe , “Ex‐Vivo Lung Perfusion,” Current Opinion in Anesthesiology 33, no. 1 (2020): 50–54. 31688085 10.1097/ACO.0000000000000804

[3] A. T. Sage , L. L. Donahoe , A. A. Shamandy , et al., “A Machine‐Learning Approach to Human Ex Vivo Lung Perfusion Predicts Transplantation Outcomes and Promotes Organ Utilization,” Nature Communications 14, no. 1 (2023): 4810, 10.1038/s41467-023-40468-7. PMC10412608 37558674

[4] M. Cypel , J. C. Yeung , S. Hirayama , et al., “Technique for Prolonged Normothermic Ex Vivo Lung Perfusion,” The Journal of Heart and Lung Transplantation 27, no. 12 (2008): 1319–1325, 10.1016/j.healun.2008.09.003. 19059112

[5] D. Tian , Y. Wang , H. Shiiya , et al., “Outcomes of Marginal Donors for Lung Transplantation after Ex Vivo Lung Perfusion: A Systematic Review and Meta‐Analysis,” The Journal of Thoracic and Cardiovascular Surgery 159, no. 2 (2020): 720–730.e6, 10.1016/j.jtcvs.2019.07.087. 31548078

[6] M. Di Nardo , L. Del Sorbo , A. Sage , et al., “Predicting Donor Lung Acceptance for Transplant during Ex Vivo Lung Perfusion: The EX Vivo Lung PerfusIon PREdiction (EXPIRE),” American Journal of Transplantation 21, no. 11 (2021): 3704–3713. 33872459 10.1111/ajt.16616

[7] A. T. Sage , M. Richard‐Greenblatt , K. Zhong , et al., “Prediction of Donor Related Lung Injury in Clinical Lung Transplantation Using a Validated Ex Vivo Lung Perfusion Inflammation Score,” The Journal of Heart and Lung Transplantation 40, no. 7 (2021): 687–695, 10.1016/j.healun.2021.03.002. 33781664

[8] T. M. Egan , B. E. Haithcock , J. Lobo , et al., “Donation after Circulatory Death Donors in Lung Transplantation,” Journal of Thoracic Disease 13, no. 11 (2021): 6536–6549, 10.21037/jtd-2021-13. 34992833 PMC8662509

[9] S. Okahara , B. Levvey , M. McDonald , et al., “A Retrospective Review of Declined Lung Donors: Estimating the Potential of Ex Vivo Lung Perfusion,” The Annals of Thoracic Surgery 112, no. 2 (2021): 443–449, 10.1016/j.athoracsur.2020.08.042. 33121967

[10] J. E. van Zanden , H. G. D. Leuvenink , E. A. M. Verschuuren , M. E. Erasmus , and M. C. Hottenrott , “A Translational Rat Model for Ex Vivo Lung Perfusion of Pre‐Injured Lungs after Brain Death,” PLoS ONE 16, no. 12 (2021): 0260705. 10.1371/journal.pone.0260705 PMC8638921 34855870

[11] G. A. Parrilla , W. R. Hunt , and M. A. Daneshmand , “Lung Transplantation Following Donation after Circulatory Death,” Transplantation Reports 7, no. 4 (2022): 100110, 10.1016/j.tpr.2022.100110.

[12] T. Watanabe , M. Cypel , and S. Keshavjee , “Ex Vivo Lung Perfusion,” Journal of Thoracic Disease 13, no. 11 (2021): 6602–6617, 10.21037/jtd-2021-23. 34992839 PMC8662477

[13] A. K. Israni , D. A. Zaun , K. Gauntt , et al., “OPTN/SRTR 2021 Annual Data Report: Deceased Organ Donation,” American Journal of Transplantation 23, no. 2 (2023): S443–S474, 10.1016/j.ajt.2023.02.010. 37132344

[14] M. Cypel , J. C. Yeung , M. Liu , et al., “Normothermic Ex Vivo Lung Perfusion in Clinical Lung Transplantation,” New England Journal of Medicine 364, no. 15 (2011): 1431–1440, 10.1056/NEJMoa1014597. 21488765

[15] M. Cypel , J. C. Yeung , T. Machuca , et al., “Experience with the First 50 Ex Vivo Lung Perfusions in Clinical Transplantation,” The Journal of Thoracic and Cardiovascular Surgery 144, no. 5 (2012): 1200–1207, 10.1016/j.jtcvs.2012.08.009. 22944089

[16] D. Nakajima , M. Cypel , R. Bonato , et al., “Ex Vivo Perfusion Treatment of Infection in Human Donor Lungs,” American Journal of Transplantation 16, no. 4 (2016): 1229–1237, 10.1111/ajt.13562. 26730551

[17] T. Koike , J. C. Yeung , M. Cypel , et al., “Kinetics of Lactate Metabolism during Acellular Normothermic Ex Vivo Lung Perfusion,” The Journal of Heart and Lung Transplantation 30, no. 12 (2011): 1312–1319, 10.1016/j.healun.2011.07.014. 21930395

[18] C. Divithotawela , M. Cypel , T. Martinu , et al., “Long‐Term Outcomes of Lung Transplant with Ex Vivo Lung Perfusion,” JAMA Surgery 154, no. 12 (2019): 1143–1150, 10.1001/jamasurg.2019.4079. 31596484 PMC6802423

[19] P. G. Chan , A. Kumar , K. Subramaniam , and P. G. Sanchez , “Ex Vivo Lung Perfusion: A Review of Research and Clinical Practices,” Seminars in Cardiothoracic and Vascular Anesthesia 24 (2020): 34–44. 32036756 10.1177/1089253220905147

[20] A. Lyengar , A. Schiazza , and E. Cantu , “Ex‐Vivo Lung Perfusion Therapies: Do They Add Value to Organ Donation?,” Current Opinion in Organ Transplantation 27, no. 3 (2022): 204–210, 10.1097/MOT.0000000000000961. 35649110 PMC9160442

[21] J. Todd , A. Gepp , B. Richards , and B. J. Vanstone , “Improving Mortality Models in the ICU with High‐Frequency Data,” International Journal of Medical Informatics 129 (2019): 318–323, 10.1016/j.ijmedinf.2019.07.002. 31445273

[22] H.‐C. Thorsen‐Meyer , A. B. Nielsen , A. P. Nielsen , et al., “Dynamic and Explainable Machine Learning Prediction of Mortality in Patients in the Intensive Care Unit: A Retrospective Study of High‐Frequency Data in Electronic Patient Records,” The Lancet Digital Health 2, no. 4 (2020): e179–e191, 10.1016/S2589-7500(20)30018-2. 33328078

[23] F. Valenza , L. Rosso , M. Pizzocri , et al., “The Consumption of Glucose during Ex Vivo Lung Perfusion Correlates with Lung Edema,” Transplantation Proceedings 43, no. 4 (2011): 993–996, 10.1016/j.transproceed.2011.01.122. 21620034

[24] A. J. Trindade , C. T. Demarest , J. W. Stokes , et al., “Outcomes Associated with Remote, Centralized Ex Vivo Lung Perfusion (Rc‐EVLP) for Donor Lungs in a Real‐World Setting,” JTCVS Open 26 (2025): 292–298, 10.1016/j.xjon.2025.04.002. 40923083 PMC12414424

[25] R. Lin , F. Brown , S. James , J. Jones , and E. Ekinci , “Continuous Glucose Monitoring: A Review of the Evidence in Type 1 and 2 Diabetes Mellitus,” Diabetic Medicine 38, no. 5 (2021): 14528, 10.1111/dme.14528. 33496979

[26] O. Djassemi , O. Vila Dieguez , M. Reynoso , et al., “Continuous Lactate Monitoring for Real‐Time Fatigue Assessment in Baseball Pitchers,” Biosensors and Bioelectronics 304 (2026): 118611, 10.1016/j.bios.2026.118611. 41833085

[27] F. Bakhshandeh , H. Zheng , N. G. Barra , et al., “Wearable Aptalyzer Integrates Microneedle and Electrochemical Sensing for in Vivo Monitoring of Glucose and Lactate in Live Animals,” Advanced Materials 36, no. 35 (2024): 2313743, 10.1002/adma.202313743. 38752744

[28] A. T. Mazzeo , V. Fanelli , M. Boffini , et al., “Feasibility of Lung Microdialysis to Assess Metabolism during Clinical Ex Vivo Lung Perfusion,” The Journal of Heart and Lung Transplantation 38, no. 3 (2019): 267–276, 10.1016/j.healun.2018.12.015. 30642797

[29] H. Abouali , S. Srikant , M. F. A. Fattah , et al., “A Bead‐Based Quantum Dot Immunoassay Integrated with Multi‐Module Microfluidics Enables Real‐Time Multiplexed Detection of Blood Insulin and Glucagon,” Advanced Science 12, no. 29 (2025): 2412185, 10.1002/advs.202412185. 40278502 PMC12362770

[30] M. Poudineh , C. L. Maikawa , E. Y. Ma , et al., “A Fluorescence Sandwich Immunoassay for the Real‐Time Continuous Detection of Glucose and Insulin in Live Animals,” Nature Biomedical Engineering 5, no. 1 (2021): 53–63, 10.1038/s41551-020-00661-1. PMC7856282 33349659

[31] H. Aghamohammadi , K. E. Thomas , S. Srikant , J. Deglint , A. Wong , and M. Poudineh , “A Competitive, Bead‐Based Assay Combined with Microfluidics for Multiplexed Toxin Detection,” Lab on a Chip 23, no. 14 (2023): 3245–3257. 37350658 10.1039/d3lc00125c

[32] H. Abouali , Hesam‐Abouali/2B‐QIRT‐ELISA: 2B‐QIRT‐ELISA (Zenodo, 2024).

[33] Y. Tan , M. Young , A. Girish , et al., “Predicting Respiratory Decompensation in Mechanically Ventilated Adult ICU Patients,” Frontiers in Physiology 14 (2023): 1125991, 10.3389/fphys.2023.1125991. 37123253 PMC10140580

[34] G. B. Rehm , I. Cortés‐Puch , B. T. Kuhn , et al., “Use of Machine Learning to Screen for Acute Respiratory Distress Syndrome Using Raw Ventilator Waveform Data,” Critical care explorations 3, no. 1 (2021): 0313, 10.1097/CCE.0000000000000313. PMC7803688 33458681

[35] J. E. Park , D. Y. Kim , J. W. Park , et al., “Development of a Machine Learning Model for Predicting Weaning Outcomes Based Solely on Continuous Ventilator Parameters during Spontaneous Breathing Trials,” Bioengineering 10, no. 10 (2023): 1163. 37892893 10.3390/bioengineering10101163 PMC10604888

[36] L. J. Kanbar , W. Shalish , C. C. Onu , et al., “Automated Prediction of Extubation Success in Extremely Preterm Infants: The APEX Multicenter Study,” Pediatric Research 93, no. 4 (2023): 1041–1049, 10.1038/s41390-022-02210-9. 35906315

[37] T. A. Nguyen , J. Matousek , A. Kubena , et al., “Ventilator Variables Predicting Extubation Readiness in Extremely Premature Infants with Prolonged Mechanical Ventilation: A Retrospective Observational Study,” Pediatric Pulmonology 59, no. 12 (2024): 3585–3592, 10.1002/ppul.27265. 39267451 PMC11600990

[38] C. L. Downey , S. Chapman , R. Randell , J. M. Brown , and D. G. Jayne , “The Impact of Continuous versus Intermittent Vital Signs Monitoring in Hospitals: A Systematic Review and Narrative Synthesis,” International Journal of Nursing Studies 84 (2018): 19–27, 10.1016/j.ijnurstu.2018.04.013. 29729558

[39] B. A. Rowland , V. Motamedi , F. Michard , A. K. Saha , and A. K. Khanna , “Impact of Continuous and Wireless Monitoring of Vital Signs on Clinical Outcomes: A Propensity‐Matched Observational Study of Surgical Ward Patients,” British Journal of Anaesthesia 132, no. 3 (2024): 519–527, 10.1016/j.bja.2023.11.040. 38135523

[40] C. D. Galloway , A. V. Valys , J. B. Shreibati , et al., “Development and Validation of a Deep‐Learning Model to Screen for Hyperkalemia from the Electrocardiogram,” JAMA Cardiology 4, no. 5 (2019): 428–436, 10.1001/jamacardio.2019.0640. 30942845 PMC6537816

[41] D. Adedinsewo , R. E. Carter , Z. Attia , et al., “Artificial Intelligence‐Enabled ECG Algorithm to Identify Patients with Left Ventricular Systolic Dysfunction Presenting to the Emergency Department with Dyspnea,” Circulation: Arrhythmia and Electrophysiology 13, no. 8 (2020): 008437. 10.1161/CIRCEP.120.008437 32986471

[42] A. A. Rabinstein , M. D. Yost , L. Faust , et al., “Artificial Intelligence‐Enabled ECG to Identify Silent Atrial Fibrillation in Embolic Stroke of Unknown Source,” Journal of Stroke and Cerebrovascular Diseases 30, no. 9 (2021): 105998, 10.1016/j.jstrokecerebrovasdis.2021.105998. 34303963

[43] F. van Wyk , A. Khojandi , A. Mohammed , E. Begoli , R. L. Davis , and R. Kamaleswaran , “A Minimal Set of Physiomarkers in Continuous High Frequency Data Streams Predict Adult Sepsis Onset Earlier,” International Journal of Medical Informatics 122 (2019): 55–62, 10.1016/j.ijmedinf.2018.12.002. 30623784

[44] P. L. S. Ohland , T. Jack , M. Mast , A. Melk , A. Bleich , and S. R. Talbot , “Continuous Monitoring of Physiological Data Using the Patient Vital Status Fusion Score in Septic Critical Care Patients,” Scientific Reports 14, no. 1 (2024): 7198, 10.1038/s41598-024-57712-9. 38531955 PMC10965972

[45] O. P. Alge , J. Pickard , W. Zhang , et al., “Continuous Sepsis Trajectory Prediction Using Tensor‐Reduced Physiological Signals,” Scientific Reports 14, no. 1 (2024): 18155, 10.1038/s41598-024-68901-x. 39103488 PMC11300462

[46] J. R. Ferdinand , M. I. Morrison , A. Andreasson , et al., “Transcriptional Analysis Identifies Potential Novel Biomarkers Associated with Successful Ex‐Vivo Perfusion of Human Donor Lungs,” Clinical Transplantation 36, no. 4 (2022): 14570, 10.1111/ctr.14570. PMC9285052 34954872

[47] A. S. I. Andreasson , L. A. Borthwick , C. Gillespie , et al., “The Role of Interleukin‐1β as a Predictive Biomarker and Potential Therapeutic Target during Clinical Ex Vivo Lung Perfusion,” The Journal of Heart and Lung Transplantation 36, no. 9 (2017): 985–995, 10.1016/j.healun.2017.05.012. 28551353 PMC5578478

[48] A. S. I. Andreasson , D. M. Karamanou , C. S. Gillespie , et al., “Profiling Inflammation and Tissue Injury Markers in Perfusate and Bronchoalveolar Lavage Fluid during Human Ex Vivo Lung Perfusion,” European Journal of Cardio‐Thoracic Surgery 51, no. 3 (2017): 577–586. 28082471 10.1093/ejcts/ezw358 PMC5400024

[49] A. Shrivastava and V. B. Gupta , “Methods for the Determination of Limit of Detection and Limit of Quantitation of the Analytical Methods,” Chronicles of Young Scientists 2, no. 1 (2011): 21–25, 10.4103/2229-5186.79345.

