# Supplementary material for: MetaboSense: An Integrated Quantum Dot‐Mediated Aptamer Assay and Modular Microfluidic Platform for Continuous In‐Line Monitoring of Lung Metabolism During Ex Vivo Perfusion
Source: Adv Sci (Weinh). 2026 Aug 13:e77123. Online ahead of print. doi: 10.1002/advs.77123 (PMC13472114; doi:10.1002/advs.77123)
Supplement: Supplementary file 1 [file ADVS-9999-e77123-s001.docx]

**Supplementary Information**

**MetaboSense: An Integrated Quantum Dot-Mediated Aptamer Assay and Modular Microfluidic Platform for Continuous In-Line Monitoring of Lung Metabolism during Ex Vivo Perfusion**

Sanjana Srikant MSc^1,2+^, Pouyan Keshavarz Motamed PhD^1,2+^,Thomas Borrillo MHSc^3,4^, , Lielle Ronen BSc^3-5^, Gabriel Siebiger MD^3-5^, Guillermo Garza MD^3,4^, Hesam Abouali PhD^1,2^, Md Fahim Al Fattah MSc^1,2^, Ines Jaber^3^, Dayan Ban PhD^1,2^, Marcelo Cypel MD^3-6^, Mingyao Liu MD^3-6^, Shaf Keshavjee MD^3-6^, Andrew T. Sage^3-6^* PhD and Mahla Poudineh^1,2^* PhD

1. Department of Electrical and Computer Engineering, University of Waterloo, Waterloo, ON, Canada
2. Waterloo Institute of Nanotechnology, University of Waterloo, Waterloo, ON, Canada
3. Latner Thoracic Research Laboratories, Toronto General Hospital, University Health Network, Toronto, ON, Canada
4. Toronto Lung Transplant Program, Ajmera Transplant Centre, University Health Network, Toronto, ON, Canada
5. Institute of Medical Science, Temerty Faculty of Medicine, University of Toronto, Toronto, ON, Canada
6. Department of Surgery, Temerty Faculty of Medicine, University of Toronto, Toronto, ON, Canada

+ These authors contributed equally.

*Corresponding authors:

| Mahla Poudineh, PhD | Andrew T. Sage, PhD |
| --- | --- |
| Canada Research Chair in Health Monitoring BioNano Devices | Toronto General Hospital  University Health Network |
| 200 University Ave W | 101 College St., PMCRT 2-816 |
| Waterloo, ON N2L 3G1 Canada | Toronto, ON M5G 1L7 Canada |
| Telephone: 647-781-8178 | Telephone: 416-634-7701 |
| [mahla.poudineh@uwaterloo.ca](mailto:mahla.poudineh@uwaterloo.ca) | [andrew.sage@uhn.ca](mailto:andrew.sage@uhn.ca) |

**Figure S1. Benchtop BQA assay assessment in perfusate.** i) shows the benchtop calibration curve of glucose and ii) shows the benchtop calibration curve of lactate in perfusate. FI is the fluorescence signal from beads incubated with target, and F_0_ is the signal from beads incubated with unspiked perfusate. Data shown here is the normalized signal gain value obtained from the absolute value of the signal loss of the mean of three replicates and the +/- standard deviation. This study shows the initial success of the individual assays in the capture and detection of targets in perfusate.

**Figure S2. MetaboSense microfluidic modular characterization.** A) Depicts the mixer characteristics and microscopy images of serpentine channel and herringbone grooves to facilitate chaotic mixing. The magnified u-bend on the right panel of the table shows a single serpentine channel with the embedded herringbone structures and the measurements that define these characteristics. B) The depletion module characteristics and dimensional measurements are shown along with their microscopic features. The device consists of microposts that are arranged in rows in a such a way that achieves separation of particles by size according to criteria previously optimized and described. The size of the posts, their horizontal and vertical gaps, the number of rows, and posts per row, are designed to achieve a critical diameter of 14 um in this work, which means that any particles larger than 14 um – beads that are conjugated to aptamer-displacement strand-quantum dot complex, and the captured targets – are flown towards the bead outlet, and particles that are smaller are separated out toward the waste outlet – these would include unbound displacement strand-quantum dot complex, perfusate components, and stray hematocrit. C) Depicts the detection module with it’s sample channel, shown as the horizontal channel, the excitation groove shown as the vertical channel, and the two collection grooves shown as the two angled channels on either side of the excitation channel. The dimensions are discussed in the left panel.

**Figure S3. Control experiments to validate aptamer-dependent signal generation.** Microbeads functionalized with competitor strands in the absence of aptamer probes were incubated with the lowest (0 mM) and highest (20 mM) target analyte concentrations. Fluorescence intensity (FI) was measured using a plate reader. Data represent the mean ± SD of three independent replicates; comparisons are done via unpaired t-test.‘ns’ indicates no significant difference (p > 0.7 for Glucose, and p > 0.3 for Lactate), confirming that signal generation is strictly dependent on the presence of aptamer probes.

**Figure S4.** Continuous operation of the MetaboSense platform over 2 hours at a constant lactate concentration of 10 mM. The platform maintained stable measurements, with device replacements conducted at 30 and 75 minutes to mitigate signal overshoot attributed to bead aggregation in the depletion module.

**Figure S5. In-line EVLP MetaboSense characterization.** i) Bead recovery efficiency within the integrated MetaboSense platform during EVLP circuit operation. Recovery was determined by quantifying beads at the detection module outlet versus those collected in the depletion module waste outlet via flow cytometry. ii) Depletion performance of the integrated system. Assessment was performed by injecting FITC-conjugated DNA into the device while connected to the EVLP circuit; depletion efficiency was calculated by comparing fluorescence levels in the final device outlet versus the depletion module waste outlet using a plate reader. iii) Glucose correlation with ABG measurements and iv) lactate correlation with ABG measurements across all in-line measurements.

**Table S1.** Glucose and lactate assay LOD and LOQ

|  | SD of blank sample | Slope of the linear calibration fit (mM^-1^) | LOD (mM) | LOQ (mM) |
| --- | --- | --- | --- | --- |
| **Glucose** | 0.024 | 0.0985 | 0.74 | 2.5 |
| **Lactate** | 0.046 | 0.1167 | 1.19 | 3.97 |

**Table S2.** Donor demographic data

|  | **MetaboSense cohort (n=11)** |
| --- | --- |
| **Donors** |  |
| Age, years | 40±19 |
| BMI | 28.7±11.6 |
| TLC (L) | 6.26±1.13 |
| Sex, n (%) |  |
| Male | 6 (55%) |
| Female | 5 (45%) |
| Donation Type, n (%) |  |
| DCD | 3 (27%) |
| DBD | 8 (73%) |
| Transplanted, n (%) | |
| Accepted | 8 (73%) |
| Declined | 3 (27%) |

BMI = body mass index; TLC = total lung capacity; DBD = donation after brain death; DCD = donation after cardiac death
